# “Neural Efficiency” of Athletes’ Brain during Visuo-Spatial Task: An fMRI Study on Table Tennis Players

**DOI:** 10.3389/fnbeh.2017.00072

**Published:** 2017-04-26

**Authors:** Zhiping Guo, Anmin Li, Lin Yu

**Affiliations:** ^1^School of Kinesiology, Shanghai University of SportShanghai, China; ^2^Neurocognition and Action—Biomechanics Research Group Center of Excellence—Cognitive Interaction Technology (CITEC), Bielefeld UniversityBielefeld, Germany

**Keywords:** neural efficiency, visuo-spatial information processing, sports training, brain activation, table tennis players, functional magnetic resonance imaging

## Abstract

Long-term training leads experts to develop a focused and efficient organization of task-related neural networks. “Neural efficiency” hypothesis posits that neural activity is reduced in experts. Here we tested the following working hypotheses: compared to non-athletes, athletes showed lower cortical activation in task-sensitive brain areas during the processing of sports related and sports unrelated visuo-spatial tasks. To address this issue, cortical activation was examined with fMRI in 14 table tennis athletes and 14 non-athletes while performing the visuo-spatial tasks. Behavioral results showed that athletes reacted faster than non-athletes during both types of the tasks, and no accuracy difference was found between athletes and non-athletes. fMRI data showed that, athletes exhibited less brain activation than non-athletes in the bilateral middle frontal gyrus, right middle orbitofrontal area, right supplementary motor area, right paracentral lobule, right precuneus, left supramarginal gyrus, right angular gyrus, left inferior temporal gyrus, left middle temporal gyrus, bilateral lingual gyrus and left cerebellum crus. No region was significantly more activated in the athletes than in the non-athletes. These findings possibly suggest that long-standing training prompt athletes develop a focused and efficient organization of task-related neural networks, as a possible index of “neural efficiency” in athletes engaged in visuo-spatial tasks, and this functional reorganization is possibly task-specific.

## Introduction

Extensive practice over a long period of time leads expert athletes to develop a focused and efficient organization of task-related neural networks (Milton et al., 2007), and the functional reorganization is task-specific rather than general in terms of improved motor abilities (Schwenkreis et al., 2007). “Neural efficiency” hypothesis posits that neural activity is reduced in experts (Del Percio et al., 2009a). Present studies investigating expert athletes’ specific brain activation are somewhat inconsistent.

Numerous previous studies showed that compared to novices/non-athletes, expert athletes have less brain activation during resting state or performing cognitive/motor tasks. For example, in the condition of resting state, karate athletes exhibited less cortical activation over frontal, central, parietal or occipital areas than non-athletes (Babiloni et al., 2010a; Del Percio et al., 2011b). During viewing pictures/videos of real competition performances, alpha event-related desynchronization (ERD) was lower in mirror system in athletes than in non-athletes (Babiloni et al., 2009, 2010b). During the 6 s pre-shot period, athletes exhibited greater alpha power than novices in occipital areas (Loze et al., 2001), parietal (Baumeister et al., 2008) and the whole scalp (Del Percio et al., 2009b). Besides, compared to non-athletes and skilled athletes, elite athletes showed lower coherence values, which imply the refinement of cortical networks in experts and differences in strategic planning related to memory processes and executive influence over visual-spatial cues (Deeny et al., 2009). During the execution of upright standing, less alpha ERD was observed in frontal, central and parietal areas in athletes (Del Percio et al., 2009a). Similar results were observed in primary motor area, lateral and medial premotor areas in athletes while performing wrist extension task (Del Percio et al., 2010).

However, many other studies reported more, or partly, cortical activation in expert athletes than in non-athletes. For instance, alpha power in athletes was reduced significantly (more cortical activation) while they observed sports videos, which was not found in novices (Orgs et al., 2008). Besides, a TMS study observed greater activation in the frontal mirror system in athletes than in novices during observation of sports videos (Aglioti et al., 2008), and two fMRI studies also observed greater activation in task related brain areas in athletes than in novices/non-athletes while they observed sports videos (Wright et al., 2011) or judged the line orientation (Seo et al., 2012). In addition, during preparing or executing a motor task, athletes exhibited higher alpha coherence values in parietal, temporal and occipital areas (Del Percio et al., 2011a) or more alpha ERD in ventral centro-parietal pathway than novices (Del Percio et al., 2007a). It’s worth noting that a few fMRI studies examined the effect of task familiarity on athletes’ brain activation and found greater cortical activation in task-sensitive areas (e.g., the mirror system, motor areas) in athletes while performing familiar tasks than less familiar tasks (Calvo-Merino et al., 2005, 2006; Lyons et al., 2010; Woods et al., 2014). These different findings might be related to practice-related decrease (mainly in frontal cortex areas), increase (mainly in task-relevant brain areas), redistribution and reorganization of regional activation of cognitive and sensorimotor processes (Kelly and Garavan, 2005; Babiloni et al., 2010b; Hardwick et al., 2013).

Considering the inconsistent results of the brain activation in athletes, most of these studies employed motor or motor related tasks and few studies adopted cognitive tasks, the present fMRI study contributed to the debate on the more or less brain activation in athletes during cognitive tasks. The cortical activation was examined when athletes and non-athletes performed visuo-spatial tasks. Based on the “Type Token Model” (Zimmer and Ecker, 2010) and the item characteristic of table tennis, we used a visuo-spatial task that included sports related condition and sports unrelated condition, in which participants were asked to recognize the figure (circle or cross-star) with notch angle of 135°. The following hypothesis was tested in the present study: athletes exhibited lower cortical activation in task-sensitive brain areas than non-athletes during processing of sports related and sports unrelated visuo-spatial task. The ventral and dorsal cortical visual pathways were considered as they were respectively involved in the recognition of objects (Braddick and Atkinson, 2007) and the analysis of visual space (Rolls and Stringer, 2006). In addition, after reviewing studies from functional and structural neuroimaging paradigms, Jung and Haier (2007) report a striking consensus suggesting that variations in a distributed network predict individual differences found on intelligence and reasoning tasks, and they describe this network as the Parieto-Frontal Integration Theory (P-FIT). According to the P-FIT, the extrastriate cortex, fusiform gyrus, supramarginal, superior parietal, angular gyri, frontal regions and anterior cingulated are the very critical brain areas in solving a given problem (Jung and Haier, 2007). Statistical analysis of the present study focused on the following brain areas/cortex: extrastriate cortex, fusiform gyrus, supramarginal, superior parietal, angular gyri, cingulated, frontal regions and cerebellum.

## Materials and Methods

### Participants

A total of 28 right handed male subjects, 14 table tennis players (mean age, 19.64 ± 1.50 years) and 14 non-athletes (mean age, 21.50 ± 1.83 years) participated in the experiment. None of the non-athletes had any formal table tennis training experience. All of the table tennis players were above the 2nd level of national standard and had been practicing table tennis for more than 8 years at least five times a week. All subjects reported normal or corrected vision and no history of mental disorders problems.

This study was approved by the Ethics Committee of Scientific Research of Shanghai University of Sport (no. 2014066) and carried out strictly in accordance with the approved guidelines. All participants gave informed written consent.

### Experiment Task

**Figure 1:**
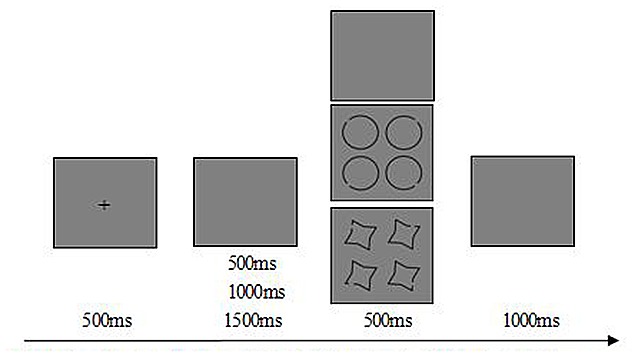
**Schematic illustration of the stimulus for one trail**. Each trails starts with a 500 ms fixation of cross on gray background. At the end of the fixation, 500 ms/1000 ms/1500 ms of a jitter will appear, and then appears the 500 ms probe stimuli. After the probe stimuli, there is 1000 ms of a gray screen for subject’s response.

The experimental task was a go/no-go visuo-spatial task. “Type Token” model, a theoretical model of long-term object memory, suggesting that perceptual priming and episodic recognition are phenomena based on distinct kinds of representations, i.e., types and tokens. Types are prototypical representations needed for object identification, mainly include the outline and three-dimension information. Tokens support episodic recognition, mainly store the orientation and color information, and the tokens can be bound preserved with types. Individuals can simplify the types and tokens to form a special bundled representation for a long time of contacting with some objects (Zimmer and Ecker, 2010). Based on the “Type Token” model and the item characteristic of table tennis, circle with notch angle over 45°, 135°, 225°or 315° was employed as sport related stimulus for its similarity on the ball and the hitting point (Zhang, 2014). The cross-star with notch angle over 45°, 135°, 225° or 315° was employed as sport unrelated stimuli for its shape’s unfamiliarity in table tennis. The target stimulus was the shape with notch angle over 135° and only appeared at one location of the picture (There were four shapes in one picture). The ratio of the target and non-target stimulus is 50%, respectively. Participants were asked to press the left key with right index finger when the circle target stimulus displayed, press the right key with the right third finger when the cross-star target stimulus showed, and instructed not to press key while non-target stimulus displayed. All stimuli appeared in a pseudorandom order. The total number of trials was 256, 60 go trials for circle and cross-star respectively, 60 no-go trials for circle and cross-star trials respectively, 16 no-go trials for blank screen as baseline. The schematic illustration of the stimulus for one trail was shown in Figure 1.

### Image Acquisition/Scanning Parameters

fMRI scanning was conducted using a Siemens Magnetom Verio 3T MRI scanner and a 32-channel head coil. Functional data consisted of 384 volumes using a T_2_-weighted echo planar imaging sequence with 33 contiguous sagittal slices covering the whole brain. The data was acquired with an FOV of 220 × 220 mm, flip angle 90°, TR of 2000 ms, TE of 30 ms and slice thickness of 3 mm. The resulting voxel resolution was 3.4 × 3.4 × 3.0 mm.

Participants indicated their judgment by pressing one of two buttons of an MRI-compatible response device held in the right hand (left button for sport related go stimuli and right button for sport non-related go stimuli).

### Image Analysis

Image processing and statistical analyses were based on MATLAB (The Mathworks Inc., Natick, MA, USA, release 9) and SPM12 (SPM; Wellcome Department of Imaging Neuroscience, London, UK; online at http://www.fil.ion.ucl.ac.uk), and the result was visualized using xjView toolbox (online at http://www.alivelearn.net/xjview). Preprocessing included realignment, slice-time correction and normalization to the standard space of the Montreal Neurological Institute brain (MNI brain). Smoothing was conducted with an isotropic three-dimensional Gaussian filter with a full-width-at-half-maximum kernel (FWHM) of 6 mm. The functional images were corrected for sequential slice timing, and all images were realigned to the middle image to correct for head movement between scans. The realigned images were then mean-adjusted by proportional scaling and spatially normalized into standard stereotactic space to fit a MNI template based on the standard coordinate system.

The pre-processed fMRI data were then entered into first-level individual analysis by comparing fMRI activity during the target stimuli presenting condition (sport related and sport unrelated condition) with that during the blank presenting condition (baseline condition).

In second-level analysis, contrast images from the analysis of individual subjects were analyzed by a 2 (Group: Athletes, Non-athletes) × 2 (Stimulus Type: Sports related, Sports unrelated) ANOVA (with Group as a between-subjects factor and Stimulus Type as a within-subjects factor). Regions showing a significant interaction were identified using an initial uncorrected voxel-wise threshold of *F*_(1,52)_ = 12.164, *p* < 0.001.

### Analysis of Behavioral Data

Repeated ANOVA was used to check the reaction time and accuracy differences between athletes and non-athletes among sports related and unrelated stimulus.

## Results

### Behavioral Results

The behavioral outcomes (task accuracy and response time) were shown in Table 1. A 2 × 2 repeated ANOVA was used to determine group differences for behavioral outcomes, employing the SPSS software. Statistical significance was defined at *p* < 0.05.

**Table 1:** **Behavioral measurement of athletes and non-athletes under sports related task and sports unrelated task**.

Group	Stimulus type	Accuracy (%)	Reaction time (ms)
		Mean ± SD	Mean ± SD
Athletes	Sports related	80.14 ± 7.74	682.25 ± 57.52
(*n* = 14)	Sports unrelated	76.71 ± 8.71	691.57 ± 54.30
Non-athletes	Sports related	79.79 ± 9.86	744.02 ± 68.01
(*n* = 14)	Sports unrelated	77.36 ± 10.63	770.23 ± 75.96

The ANOVA of the accuracy variable showed no statistical significant differences in main effect or interaction between the factors Group (athletes, non-athletes) and Condition (Sports related, Sports unrelated; *p* > 0.05). The ANOVA of the reaction time showed no statistically significant differences in interaction between the factors Group (athletes, non-athletes) and Condition (Sports related, Sports unrelated; *p* > 0.05), but displayed significant differences in main effect between groups (*F*_(1,52)_ = 10.05, *p* = 0.004, *η*^2^ = 0.279). Compared with non-athletes, athletes needed much less time to recognize the target stimulus during both sports related task and sports unrelated task.

### Imaging Results

A few regions showed a significant Expertise × Stimulus-Type interaction at the whole-brain level, including lingual gyrus (BA 18), cuneus (BA 19), superior occipital lobe (BA 19), supramarginal (BA 40), cingulate gyrus (BA 24), paracentral lobule/ precuneus (BA 5), supplemental motor area (BA 6), medial superior frontal gyrus (BA 8). The *post hoc* tests were then used to check the simple effect for different factor level.

#### Group Effect under Sports Related Stimulus Condition

**Figure 2:**
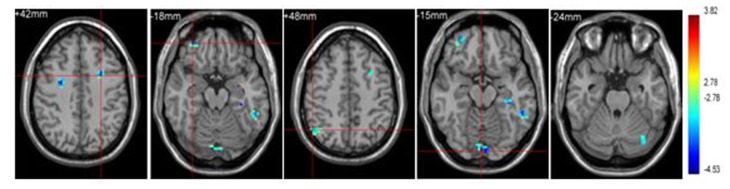
**Brain regions activated in “sports related condition” from between group analysis (*p* < 0.001, uncorrected, cluster size of 15)**.

Significant brain regions of group effect under sports related stimulus condition were shown in Figure 2 and Table 2.

**Table 2:** **Brain regions activated in “sports related condition” from between group analysis (H = hemisphere; *p* < 0.001, uncorrected)**.

Group	Region	H	Cluster size	MNI coordinates			Peak *T*
				*x*	*y*	*z*
Athletes < Non-athletes	Frontal_Mid_L (aal) (BA6)	L	25	−27	6	42	3.97
	Frontal_Mid_Orb_R (aal) (BA 10)	R	41	33	48	−18	4.01
	Angular_R (aal) (BA 39)	R	31	51	−60	48	3.50
	Lingual Gyrus (BA 22)	R	28	3	−90	−15	4.19
	Cerebelum_Crus1_L (aal)	L	15	−33	−75	−24	3.36

Athletes exhibited less activation than non-athletes in the left middle frontal gyrus, right middle orbitofrontal area, right angular gyrus and left cerebellum crus. No region was significantly more activated in the athletes than in the non-athletes.

#### Group Effect under Sports Unrelated Stimulus Condition

**Figure 3:**
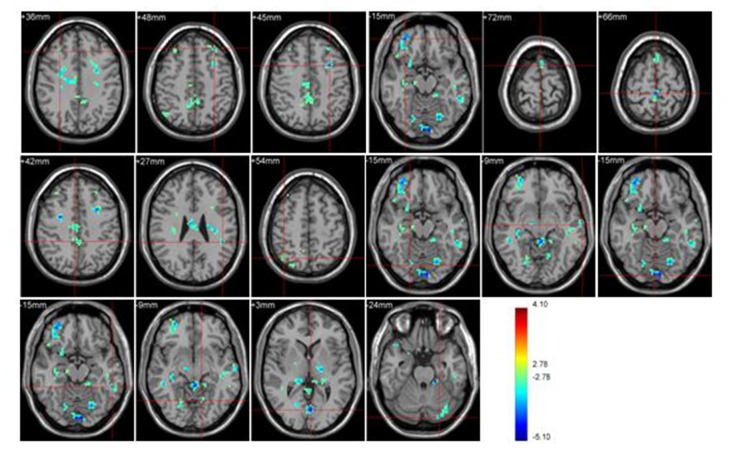
**Brain regions activated during “sports related condition” from between group analysis (*p* < 0.001, uncorrected, cluster size of 15)**.

Significant brain regions of group effect under sports unrelated stimulus condition were shown in Figure 3 and Table 3.

**Table 3:** **Brain regions activated during “sports related condition” from between group analysis (H = hemisphere; *p* < 0.001, uncorrected)**.

Group	Region	H	Cluster size	MNI coordinates			Peak *T*
				*x*	*y*	*z*	
Athletes < Non-athletes	Middle Frontal Gyrus (BA 6)	R	19	30	30	36	3.56
		L	23	−30	36	48	3.66
		L	91	−30	9	45	4.40
	Frontal_Mid_Orb_R (aal) (BA 10)	R	118	33	48	−15	4.48
	Supp_Motor_Area_L (aal) (BA 6)	L	42	0	9	72	3.48
	Paracentral Lobule (BA 31)	R	55	0	−33	66	4.41
	Precuneus (BA 7)	R	92	6	−39	42	3.38
	SupraMarginal_L (aal) (BA 40)	L	56	−45	−39	27	4.18
	Angular_R (aal) (BA 39)	R	33	39	−93	54	3.19
	Temporal_Inf_L (aal) (BA 20)	L	24	−54	−42	−15	3.95
	Middle Temporal Gyrus (BA 21)	L	18	−66	−15	−9	3.41
	Lingual Gyrus (BA 18)	R	38	3	−90	−15	4.52
		R	59	21	−75	−15	3.73
		L	18	−12	−63	−9	3.51
		L	88	−3	−75	3	4.79
	Cerebelum_Crus1_L (aal)	L	27	−24	−87	−24	3.63

Athletes exhibited less activation than non-athletes in the bilateral middle frontal gyrus, right middle orbitofrontal area, right supplementary motor area, right paracentral lobule, right precuneus, left supramarginal gyrus, right angular gyrus, left inferior temporal gyrus, left middle temporal gyrus, bilateral lingual gyrus and left cerebellum crus. No region was significantly more activated in the athletes than in the non-athletes.

#### Stimulus Type Effect under Athlete Condition

**Figure 4:**
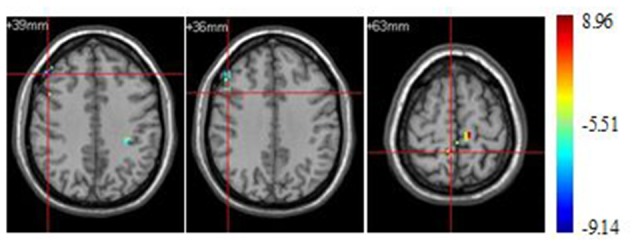
**Brain regions activated during “athlete condition” from between stimulus type analysis (*p* < 0.0001, uncorrected, cluster size of 15)**.

Significant brain regions of stimulus type effect under athlete condition were shown in Figure 4 and Table 4. The left middle frontal gyrus and pars opercularis of inferior frontal gyrus exhibited less activation under sports related condition than sports unrelated condition in athletes, but the precuneus exhibited more activation under sports related condition than sports unrelated condition in athletes.

**Table 4:** **Brain regions activated during “athlete condition” from between stimulus type analysis (H = hemisphere; *p* < 0.0001, uncorrected)**.

Stimulus type	Region	H	Cluster size	MNI coordinates			Peak *T*
				*x*	*y*	*z*	
Sports related < Sports unrelated	Frontal_Mid_R (aal) (BA 9)	R	24	48	33	39	8.46
	Frontal_Inf_Oper_R (aal) (BA 44)	R	22	48	15	36	6.65
Sports related > Sports unrelated	Precuneus_R (aal) (BA 5)	R	16	6	−45	63	8.22

#### Group Effect under Sports Unrelated Stimulus Condition

**Figure 5:**
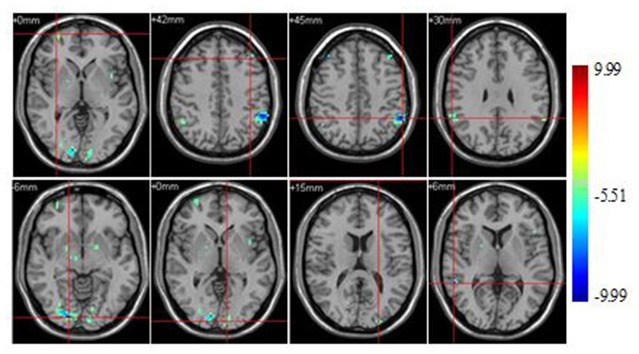
**Brain regions activated during “non-athlete condition” from between stimulus type analysis (*p* < 0.0001, uncorrected, cluster size of 15)**.

Significant brain regions of stimulus type effect under non-athlete condition were shown in Figure 5 and Table 5.

**Table 5:** **Brain regions activated during “non-athlete condition” from between stimulus type analysis (H = hemisphere; *p* < 0.0001, uncorrected)**.

Stimulus type	Region	H	Cluster size	MNI coordinates			Peak *T*
				*x*	*y*	*z*	
Sports related < Sports unrelated	Frontal_Sup_R (aal) (BA 10)	R	30	33	63	0	6.48
	Frontal_Mid_L (aal) (BA 9)	L	16	−39	30	42	7.71
	Inferior Parietal Lobule (BA 40)	L	115	−57	−48	45	9.99
	SupraMarginal_R (aal) (BA 40)	R	68	60	−48	30	6.92
	Lingual_R (aal) (BA 17)	R	124	18	−90	−6	9.09
	Occipital_Mid_L (aal) (BA 17)	L	79	−9	−96	0	6.64
		L	21	−24	96	15	7.28
	Temporal_Mid_R (aal) (BA 22)	R	20	57	−45	6	8.91

A few brain areas exhibited less activation under sports related condition than in sports unrelated condition, including the superior frontal gyrus, middle frontal gyrus, occipital lobe, inferior parietal lobule, supramarginal gyrus, lingual gyrus, middle occipital lobe and middle temporal gyrus. No region was significantly more activated under sports related condition than in sports unrelated condition.

## Discussion

This study used fMRI to investigate the brain activation in athletes and non-athletes during a figure recognition task. Our hypothesis was based on research demonstrating that athletes seems to develop a focused and efficient organization of task-related neural networks (Milton et al., 2007), the functional reorganization is task-specific rather than general in terms of improved motor abilities (Schwenkreis et al., 2007), and the “neural efficiency” hypothesis about experts (Del Percio et al., 2009a). More specifically, it was tested whether there was less cortical activity in athletes than in non-athletes during the sports related and sports unrelated visual-spatial task.

Behaviorally, we found that athletes showed shorter reaction time during both tasks than non-athletes. This result was supported by the previous findings that athletes exhibited faster than non-athletes during reaction time tasks, and the faster responses stimulus discrimination and response selection ability possibly due to athletes’ enhanced attention and inhibitory control ability (Hung et al., 2004; Di Russo et al., 2006; Nakamoto and Mori, 2008, 2012; Muraskin et al., 2015).

Regarding the group effect, neuroimaging data demonstrated less brain activation in numerous areas in athletes than in non-athletes during the visuo-spatial tasks, no brain area showed more activation in athletes than in non-athletes during either of the tasks. Less brain activation areas in athletes than in non-athletes including the bilateral middle frontal gyrus (BA 6), right middle orbitofrontal area (BA 10), right supplementary motor area (BA 6), right paracentral lobule (BA 31), right precuneus (BA 7), left supramarginal gyrus (BA 40), right angular gyrus (BA 17), left inferior temporal gyrus (BA 20), left middle temporal gyrus (BA 21), bilateral lingual gyrus (BA 18) and left cerebellum crus. These results are in line with the findings of previous research that athletes exhibited less cortical activation during social cognition task. The activation in occipital areas was decreasing in non-athletes, amateur karate athletes and elite karate athletes during the observation of pictures with basket and karate attacks (Del Percio et al., 2007b). Low-and high-frequency alpha ERD was lower in amplitude in the elite rhythmic gymnasts compared to the non-gymnasts in occipital and temporal areas (ventral pathway) and in dorsal pathway, these results globally suggest that the judgment of observed sporting actions is related to low amplitude of alpha ERD, as a possible index of spatially selective cortical activation (“neural efficiency”; Babiloni et al., 2009). Low- and high-frequency alpha ERD was less pronounced in dorsal and “mirror” pathways in the elite karate athletes than in the non-athletes during the judgment of karate actions, and the researchers concluded that less pronounced alpha ERD in athletes hints at “neural efficiency” in experts engaged in social cognition (Babiloni et al., 2010b). In addition, extensive practice over a long period of time leads experts to develop a focused and efficient organization of task-related neural networks (Milton et al., 2007). It appears that the involvement of the executive functions associated with frontal pathways decreases while the role of specialized posterior brain regions becomes more important when individuals are sufficiently trained in a cognitive task (Neubauer and Fink, 2009). Less brain activation in athletes in present study may indicate that athletes have developed focused and efficient organization of task-related neural networks and needed less supervisory control while processing visuo-spatial information, and therefore exhibited “neural efficiency” during sports related and sports unrelated visuo-spatial tasks. In addition, this functional reorganization is possible not only for task-specific but also general cognitive task.

According to the P-FIT, the visual information was first processed in temporal and occipital lobes (mainly BAs 18, 19, 37), including recognition and subsequent imagery and/or elaboration of visual input, then this basic sensory/perceptual processing is fed forward to the parietal cortex (mainly BAs 40, 7, 39), wherein completed structural symbolism, abstraction, and elaboration emerge, and the parietal cortex interacts with frontal regions (mainly BAs 6, 9, 10, 45–47) at the same time, which serve to generate various solutions to a given problem. Once the best solution is arrived up on, the anterior cingulate (BA 32) is engaged to constrain response selection and inhibit other competing responses (Jung and Haier, 2007). Less brain activation in brain areas including BAs 17, 18, 20, 21, BAs 7, 31, 40 and BAs 6 and 10 in athletes than in non-athletes during the visual-spatial tasks may suggest that athletes showed “neural efficiency” during the whole information processing flow, including the early processing of sensory information, the next information integration, the information matching identification and the last response selection procedure during these task.

Regarding the stimulus type effect, neuroimaging data demonstrated less brain activation under the sports related stimulus condition than the sports unrelated stimulus condition in both athletes and non-athletes, except for the precuneus which showed more activation under the sports related condition than the sports unrelated stimulus condition in athletes. Precuneus is involved in integration of external and internal information, and can extract information from internal memory storage according to external stimuli (Ren, 2010), the increased precuneus activation in athletes during sports related stimulus tasks possibly suggest that the processing of sports related stimulus information was based more on the athletes’ sports experience compared to non-athletes.

Analyses of combined data show that results support our hypotheses. Athletes showed less brain activation during both the sports related and sports unrelated tasks. These findings are in accordance with previous studies reporting “neural efficiency” in athletes (Del Percio et al., 2008; Babiloni et al., 2010b), and this “neural efficiency” may stem from the long term training which enabled athletes to develop a focused and efficient organization of task-related neural networks, and this functional reorganization is possibly task-specific. However, it should be noted that we conducted a cross-sectional study and our entire corollary was based on compared outcomes for the two groups of subjects, for this reason we cannot exclude that maybe some differences already existed before practicing sports. It is possible that young people having certain basic perceptual-motor skills received positive feedback during their first attempts to practice sports and they became “athletes”, while those who were less skilled gave up when they were young and became “non-athletes”. Thus, probably both nature and experience contributed to the differences found by our research. One way to solve this problem may be carrying out a longitudinal study. Instead of studying the effects of long-term field training, other specific kinds of training can be relatively easily manipulated, such as perceptual training. Previous research has shown that perceptual training can be effective, from the behavioral point of view, for both non-athletes (Savelsbergh et al., 2010; Ryu et al., 2013) and athletes (Farrow and Abernethy, 2002; Murgia et al., 2014) in a shorter time, i.e., weeks/months. Therefore, in order to explore the exact effect of training on performance and brain activation pattern of athletes during cognitive tasks, future studies could compare the performance and the brain activation pattern of a group (of either non-athletes or athletes) before and after a period of perceptual training with those of a matched control group.

## Conclusion

In summary, we used fMRI to investigate the possible brain activation difference between athletes and non-athletes in visual-spatial tasks. We found that athletes reacted faster than non-athletes during both the sports related and sports unrelated visuo-spatial tasks. Athletes decreased activation in cortical regions important for the early processing of sensory information, the next information integration, the information matching identification and the last response selection. Taken together, our findings suggest that there is neural efficiency in athletes during visuo-spatial tasks, and this “neural efficiency” may stem from the long term training which prompt athletes to develop a focused and efficient organization of task-related neural networks, and this functional reorganization is possibly task-specific.

## Author Contributions

ZG: literature research, study design, data acquisition/analysis/interpretation, manuscript preparation/editing/revision. AL: guarantor of integrity of entire study, manuscript final version approval. LY: literature research, statistical analysis, manuscript editing.

## Conflict of Interest Statement

The authors declare that the research was conducted in the absence of any commercial or financial relationships that could be construed as a potential conflict of interest. The reviewer DR and handling Editor declared their shared affiliation, and the handling Editor states that the process nevertheless met the standards of a fair and objective review.
